# Analysis of Proximal Femoral Parameters in Adolescent Idiopathic Scoliosis

**DOI:** 10.1155/2019/3948595

**Published:** 2019-04-01

**Authors:** Máté Burkus, Ádám T. Schlégl, Kristóf József, Ian O'Sullivan, István Márkus, Miklós Tunyogi-Csapó

**Affiliations:** Department of Orthopedics, Medical School, University of Pécs, H-7623 Pécs, Akác St. 1., Hungary

## Abstract

**Background:**

Assessment of the proximal femoral parameters in adolescent idiopathic scoliosis using three-dimensional radiological image reconstructions may allow better characterization than conventional techniques.

**Methods:**

EOS 3D reconstructions of spines and femurs of 320 scoliotic patients (10-18 years old) and 350 control children lacking spinal abnormality were performed and 6 proximal femoral parameters measured.

**Results:**

Individuals with adolescent idiopathic scoliosis showed a small but statistically significant decrease in neck shaft angle (average difference=2.58°) and a higher (0.22°) femoral mechanical axis–femoral shaft angle. When the two sides were compared based on curve direction, greater changes in the neck shaft angle and femoral mechanical axis–femoral shaft angle were found on the side of the convexity.

**Conclusions:**

Patients with adolescent idiopathic scoliosis were found to have a small but significantly lower neck shaft angle and higher femoral mechanical axis–femoral shaft angle, which related to the curve direction. This is postulated to be due to mechanical compensation for altered balance and centre of gravity associated with a scoliosis deformity, although the observed difference likely has negligible clinical effect.

## 1. Introduction

Adolescent idiopathic scoliosis (AIS) is one of the most common spinal deformities and may affect as many as 1-4% of adolescents [1]. While scoliosis primarily causes deformity in the frontal plane it is a complex three-dimensional disorder affecting the entire body, including also the lower extremities. Compensatory changes in the pelvis have been previously described by several authors [2–4], and further studies have found increased asymmetry of limb length and other body parts in addition to the spine deformity. For example, Burwell et al. found the upper arm on the convex side to be longer in thoracic scoliosis, whereas the right lower extremity was longer in lumbar and thoracolumbar scoliosis [5, 6]. Normelli et al. compared 18 normal and 15 scoliotic cadavers and observed a significant difference in the length of the ribs [7]. Pelvic changes have been described with changes observed in hip rotation [8], abduction and range of motion [9], and proximal femoral bone density [10]. Observed abnormalities in the pelvis, however, are believed by some to be due to rotation rather than bony deformity [11].

On reviewing the literature, studies assessing the proximal femur appear to be scarce, with just one publication evaluating the femoral parameters in AIS found in the English language. In this paper Saji et al. found abnormalities associated with the deformity, as the neck shaft angle appeared to be on average 8° higher in patients with scoliosis [12].

Accurate description of the anatomical parameters of the lower extremities is of importance as altered bony structures can affect the biomechanics of the whole limb. Changes in the mechanical axis, even if minimal, may alter joint loading and small changes have been linked to increased knee arthritis [13], and abnormal neck shaft angles and femoral torsion specifically are independent risk factors for hip osteoarthritis [14].

However, assessing the parameters of the lower limb in children accurately can pose some difficulties. While conventional full-leg, full-spine radiographs allow us to see the extremities during active weight-bearing, they suffer from magnification artifacts and only allow measurements in two dimensions at a time. CT and MRI, while allowing more accurate, three-dimensional imaging, generally require the patient to lie supine and entail higher costs and radiation burden (in the case of CT). Since 2007 our department has been using the EOS 2D/3D system for evaluation of many patients. This bidirectional X-ray scanner takes simultaneous anteroposterior and lateral images of standing patients and uses a slit collimator to prevent vertical magnification and drastically reduce dose exposure. With the EOS scanner 3D reconstructions can also be made that have been found to be similar or superior to those of CT scout-based reconstructions, while delivering a radiation dose 800-1000 times lower [15]. The feasibility and accuracy of the equipment and the sterEOS software for assessing the vertebral column, the lower extremities, and the entire lumbar-pelvic-femoral complex in routine orthopedic practice have been confirmed in several studies [15–20].

## 2. Materials and Methods

The present study aimed to identify and characterize the changes of anatomical parameters of the proximal femur using a large sample of AIS patients from 10 to 18 years old, using high accuracy 3D EOS reconstructions. We hypothesized that the asymmetrical loading due to scoliosis affects the bony anatomy of the proximal femur.

Medical records from 879 children with scoliosis were reviewed from our outpatient radiological database between 2007 and 2018. All cases with spinal deformities due to other etiologies (therefore not AIS), previous surgical intervention on the spine, and lower limb abnormality were excluded. Any images of lower quality, in which reconstruction was not reliable due to movements on recording, missing reference points, etc., were also excluded. From the remaining patients, 320 cases were selected at random (79 males and 241 females) with an age range of 10-18 years (mean age at scan 14.71±2.31). All images were recorded with the EOS 2D/3D system during routine clinical work with orthopedic indication. Prior to imaging written consent was obtained from the patients or their legal guardians, which included consent to use of the images for later clinical research. Our retrospective case-control study was permitted by the Institutional Ethical Committee.

The control group consisted of sample cases without prior spinal abnormality from the database of our department. 1024 such examinations were performed during routine work in our clinic with suspicion of orthopedic disease including suspected scoliosis (though scoliosis was not found), muscular pain of unknown origin, or follow-up scans for benign bone lesions. From this population we randomly selected a control group of patients matching our scoliosis cases in age and sex, to form the basis of this comparative study. Finally, the control group comprised 350 individuals (85 males, 265 females), with a mean age of 14.81±2.43.

**Figure 1:**
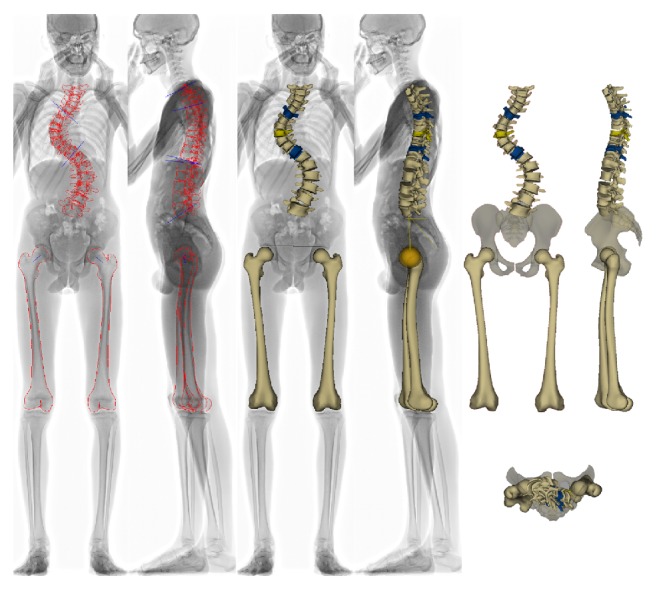
SterEOS 2D/3D spine, pelvis, and femur 3D reconstruction (from our database).

Reconstructions were made using sterEOS software EOS 3D V1.4.4.5297 (EOS Imaging, Paris, France), Cobb angles measured, and superficial 3D models of both femurs made and assessed (Figure 1).

The procedure was performed by 2 postgraduate student medical residents with considerable experience with the modality and software.

During remodeling, 30 randomly selected samples were used to assess interobserver error and intraobserver reliability of the spine reconstructions, and another 30 randomly selected 3D models of both femurs were used to assess the lower extremities on three different occasions. Intraclass coefficient was evaluated according to Winer's criteria: 0-0.24 indicating ‘absent-to-poor' reliability, 0.25-0.49 ‘low', 0.50-0.69 ‘fair to moderate', 0.7-0.89 ‘good', and 0.90-1.0 ‘excellent' [21].

**Figure 2:**
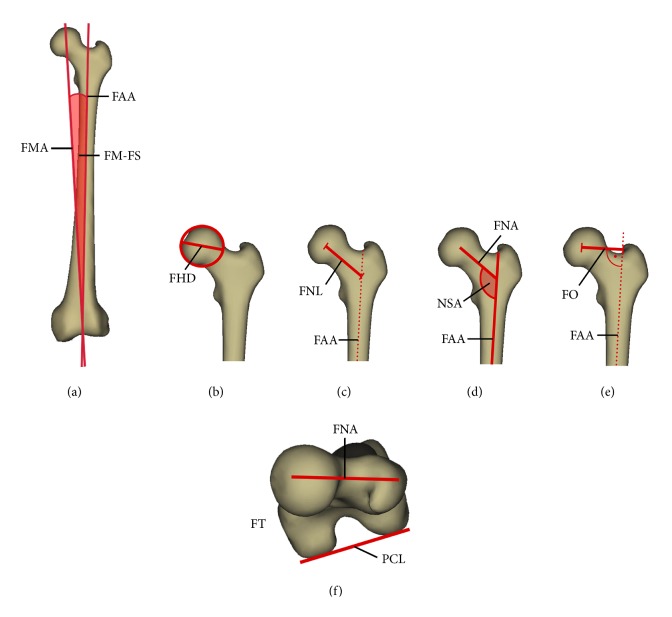
Measured parameters. (a) FM-FS: femoral mechanical axis-femoral shaft angle, FMA: femur mechanical axis, and FAA: femur anatomical axis; (b) FHD: femur head diameter; (c) FNL: femoral neck length; (d) NSA: neck shaft angle (FNA: femur neck axis); (e) FO: Femoral offset; (f) FT: femoral torsion (PCL: posterior condylar line).

Six parameters relating to the proximal femur, which are calculated during reconstruction by the sterEOS 3D software, were evaluated during the present study (neck shaft (NS) angle, femur mechanical axis-femur shaft (FM-FS) angle, femoral torsion, femoral offset, femoral head diameter, and femoral neck length) (Figure 2).

For some assessments the population was classified into four different severity groups based on the Cobb angle, using the 2011 SOSORT guidelines [22];

  Group 1: 10-25 degrees; (low or mild), n=129 (37 boys, 92 girls);
  Group 2: 25-45 degrees; (moderate), n=101 (22 boys, 79 girls);
  Group 3: 45-60 degrees; (severe), n=49 (10 boys, 39 girls);
  Group 4: over 60 degrees; (very severe), n=41 (10 boys, 31 girls).

The group distribution of sex and age is shown in Table 1.

To examine curve laterality, the population was divided into two groups based on the direction of the main scoliotic curve convexity, as the sterEOS program also defines curve direction. 151 patients had right thoracic curves and 169 had left thoracolumbar or lumbar curvatures. In the case of double curves, the laterality of the main curve was taken. Correlation between the right and left parameters and curve direction were subsequently evaluated.

The normality of values was evaluated using the Kolmogorov-Smirnov test. Differences between the mean values of the six measured parameters between control and AIS groups were evaluated using an independent sample t-test, and a one-way ANOVA was performed to assess correlation between the SOSORT severity groups and the parameters. Cobb angle parameter correlation was performed using Spearman correlation. The differences in the two sides were compared using paired t-test and regression analysis. Post-hoc power analysis was performed to control the statistical power of the t-tests. A value of p<0.05 was considered significant during our evaluation. All statistical data was processed by the SPSS v22 (IBM Corp., Armonk, NY, USA) and by the Microsoft Office Professional Plus v14.0.6112.5000 (Microsoft Corp., Redmond, WA, USA) software packages.

## 3. Results and Discussion

Intra- and interobserver reliability values for the different examiners were greater than 0.9 for each parameter, regarded as excellent as per Winer's criteria (Table 2), and proximal femur measurements were found to be normally distributed, based on the Kolmogorov-Smirnov test. The mean Cobb angle in our sample was 35.3±20.7°, with a range of 10-123 degrees.

Mean proximal femur values of the whole group of 320 scoliotic patients and 350 control sample individuals are listed in Table 3.

A higher mean FM-FS angle was found in those with AIS, whereas all other measured parameters were lower with AIS than in the control group; however most differences were not significant. The neck shaft angle was significantly lower in AIS (control 129.64±4.50°; AIS 127.06±3.85°, p<0.001, statistical power=100.0%), and the higher FM-FS angle seen was also significant (control 4.28±1.11°; AIS 4.50±0.95°, p=0.004, statistical power=78.9%). No statistically significant difference was confirmed with femoral torsion, femoral offset, femoral head diameter, and femoral neck length; however, the values were often widely spread (e.g., in femoral torsion control 20.71±9.70°; AIS 20.36±9.88°).

**Figure 3:**
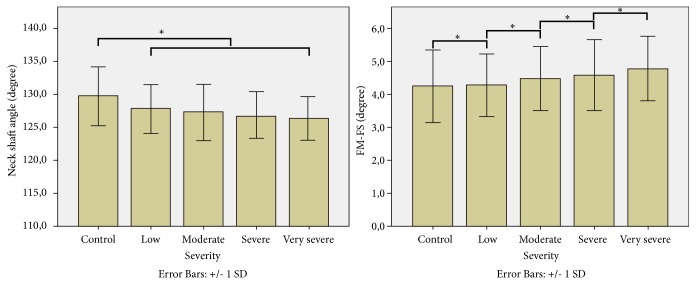
Mean values of the neck shaft angle (left) and FM-FS (right) based on the severity of the scoliosis. *∗*: significant difference based on independent sample t-test (p<0.05).

When scoliosis was stratified by severity, a significant difference was found between severity groups and the NS angle (p=0.022) and the femur mechanical-femur anatomical angle (p=0.023). Additionally, the means were compared and results are shown in Figure 3.

When the correlation between the proximal femoral parameters and curvature was performed using just the value of the Cobb angle as a continuous variable, a statistically significant value was detected in relation to the NS angle (B=-0.295, R^2^=0.87, p<0.01) and FM-FS angle (B=0.101, R^2^=0.031, p=0.002).

**Figure 4:**
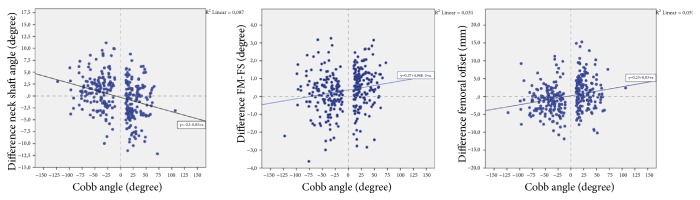
Side difference. Boxplot diagram presenting the correlation between the Cobb angle (positive values indicate a left main curve; negative values indicated right main curve) and the difference between the lower limb parameters (left side value-right side value).

With regard to laterality, when Cobb angle values and curve direction were used, it was found that the NS angle had an inverse relationship with the Cobb angle (correlation coefficient=-0.290, p<0.001), and a positive correlation with both FM-FS angle (correlation coefficient=0.191, p=0.001) and femoral offset (correlation coefficient=0.237, p<0.001) (Figure 4).

Furthermore, when means were compared, the Cobb angle had a greater effect if the convexity was left facing, than if facing to the right. The mean neck shaft angle on the side of the convexity was 1.38° lower than that of the contralateral limb in the case of left-sided curves (p=0.017, statistical power=92.2%). In individuals with right-sided curves the NS angle was 1.27° lower on the side of the curve, than the contralateral limb (p<0.001, statistical power=80.9%). The mean FM-FS angle was 0.53° higher if left-sided (p<0.001, statistical power=69.2%) and 0.13° higher if right-sided (p= 0.049, statistical power=59.2%). No significant difference was observed in the femoral offset (correlation coefficient=0.061, p=0.147), or the other values. Differences between the left and right lower limbs are presented in Table 4.

This study aimed to find and evaluate proximal femur changes that may be associated with adolescent idiopathic scoliosis. To the best of our knowledge this is the only comparative study to simultaneously evaluate this number of parameters in weight-bearing position, and in so many patients. Therefore, we aimed to make a comprehensive evaluation of the proximal femoral region and its relationship with curve severity and direction.

The present study found a small, but statistically significant, decrease in neck shaft angle in our AIS group compared to the control. This clearly contradicts what Saji et al. found in their study of Chinese women in which they analyzed 94 anteroposterior conventional X-ray images (61 with AIS and 33 control women), detecting significantly higher NS angle values in the control group (129.5±3.7° left, and 129.9±3.7° right vs. scoliosis group: 137.5±6.6° on left, and 137.9±5.5° on the right side) [12]. Their results may be related to their imaging method, which does not compensate for individual variation of the femoral anteversion, in addition to their smaller sample size. Furthermore, ethnicity may play a role as pelvic parameters found in Chinese populations have been seen to differ from those in Caucasians [2, 23–27]. The femur mechanical axis-femur shaft angle also saw a small but significant difference, with a higher value in scoliosis patients than in the control group, and this difference was seen at all severity groups and showed a significant correlation with the Cobb angle, increasing parallelly.

Interestingly the direction (convexity) of the curve was found to influence the magnitude of the change of the two parameters; if the curve is left-sided, the left NS angle was lower and FM-FS was higher, whereas if right-sided, similar changes were seen but not as great. This correlates somewhat with the findings of Chen et al. wherein the lumbar curve had significant effect on proximal femoral bone mineral density [10].

In general, both neck shaft angle and FM-FS angle exhibited similar changes that can be explained by their geometrical relationship. The axis of the neck shaft forms a triangle with the anatomical and mechanical axes of the femur, and so changes in the NS angle alter the FM-FS angle. It then becomes clear why a decrease in neck shaft angle was associated with an increase in FM-FS angle. The mean femoral offset was also elevated marginally, as would be expected from a fall in neck shaft angle without a change in femoral neck length (this marginal increase of femoral offset however was not statistically significant).

We found no statistical difference in femoral anteversion between the control group and the scoliosis group. The range of values was found to be high; however it was consistent with that of the control group findings. Previous findings for femoral anteversion by Szuper et al., obtained while studying anatomical parameters and growth factor effects, were very similar to those seen in the present study [16].

The changes seen in scoliosis patients are likely due to the altered biomechanical forces caused by a need for compensation of balance due to the deformity, or asymmetrical mechanical loading as a result of the deformity. Alterations were statistically significant, but rather small, and this minimal difference likely has no clinical effect.

AIS tends to develop during the later stages of adolescence, by which time the lower limb biomechanical parameters have been mostly established [16, 28] and hence alterations of a large magnitude are unlikely to develop. We assert that if scoliosis develops earlier, while the lower limb is undergoing more active growth, greater proximal femoral changes may be seen. However, the number of early-onset scoliosis patients is much lower than those with AIS, and even fewer among them are those who lack other systemic or generalized disorders, and so no significant group could be found in our clinical database during the period in question.

Potential limitations of this study include the control group used; individuals were not totally free of orthopedic complaints; however there are clear ethical difficulties with the exposure of 320 healthy children to ionizing radiation, even if low-dose scans are performed as with the EOS scanner. Furthermore, due to the natural incidence of AIS there was a much higher prevalence of girls in the scoliosis group, a limitation found in similar other AIS studies, and therefore we were unable to reliably evaluate sex differences in the different parameters.

## 4. Conclusions

Adolescent idiopathic scoliosis, in the absence of any other abnormalities, may have an effect on other parts of the skeleton including the proximal femur. Scoliosis patients were found to have significantly higher FM-FS angles, and significantly lower neck shaft angles, and changes exhibited a correlation with the magnitude and severity of the curve in the frontal plane. Interestingly, changes were greater if the curve convexity was left-sided. Although the observed differences were too small to conclude that AIS has an effect on the proximal femur that would be perceivable in the clinic, similar results have not been previously published.

## Figures and Tables

**Table 1:** Age and gender distribution of the examined population.

Age (year)	Low		Moderate		Severe		Very severe		Sum
	Boy	Girl	Boy	Girl	Boy	Girl	Boy	Girl	
10	5	11	1	5	1	1	0	1	*25*
11	4	10	2	4	0	3	0	2	*25*
12	3	13	1	11	0	4	0	3	*35*
13	3	9	2	11	1	6	2	6	*40*
14	6	4	4	6	1	8	1	10	*40*
15	6	9	3	9	0	6	2	5	*40*
16	1	13	3	12	4	2	4	1	*40*
17	5	14	3	9	1	5	1	2	*40*
18	4	9	3	12	2	4	0	1	*35*
	*37*	*92*	*22*	*79*	*10*	*39*	*10*	*31*	***320***

**Table 2:** Results of the intra- and interobserver reliability study, assessing reliability of EOS reconstructions. Evaluation is as per Winer's criteria.

Parameter	Intraobserver				Interobserver	
	ICC (obs 1)	ICC (obs 2)	ICC (obs 3)	Evaluation	ICC (interobs)	Evaluation
Femoral head diameter	0.95	0.93	0.97	Excellent	0.92	Excellent
Femur neck length	0.93	0.91	0.95	Excellent	0.91	Excellent
Neck shaft angle	0.96	0.96	0.95	Excellent	0.93	Excellent
Femoral offset	0.92	0.94	0.93	Excellent	0.92	Excellent
Femur mechanical axis-shaft angle	0.97	0.94	0.98	Excellent	0.94	Excellent
Femoral torsion	0.90	0.90	0.93	Excellent	0.91	Excellent
SterEOS Cobb angle	0.91	0.91	0.94	Excellent	0.90	Excellent

ICC: intraclass coefficient, obs: observer.

**Table 3:** Mean and standard deviation (SD) of the examined parameters and the results of *t*-test and ANOVA.

Parameter		Control	AIS	*t*-test (p)	Low	Moderate	Severe	Very severe	One-way Anova (p)
	*n=*	*350*	*320*	*350/320*	*129*	*101*	*49*	*41*	*320*
Age (year)	mean	14.81	14.71		14.51	15.12	15.09	14.68	
	SD	2.43	2.31		2.66	2.41	2.24	1.82	
Femoral head diam. (mm)	mean	41.89	41.66	*0.251*	41.96	41.10	41.54	41.19	*0.413*
	SD	3.76	3.62		3.65	3.79	4.02	3.56	
Femoral offset (mm)	mean	38.62	37.99	*0.098*	38.16	37.50	38.46	38.22	*0.607*
	SD	5.29	4.67		4.51	4.79	5.62	3.55	
Neck length (mm)	mean	48.51	48.11	*0.735*	49.01	47.99	48.74	47.70	*0.494*
	SD	4.67	4.76		4.68	4.95	5.36	4.23	
Neck shaft angle (degree)	mean	129.64	127.06	***>0.001***	127.49	127.09	126.61	126.09	***0.022***
	SD	4.50	3.85		3.65	4.37	3.52	3.25	
FM-FS (degree)	mean	4.28	4.50	***0.004***	4.33	4.54	4.62	4.84	***0.023***
	SD	1.11	0.95		0.92	0.93	0.98	0.99	
Femoral torsion (degree)	mean	20.71	20.36	*0.598*	20.39	20.89	20.35	19.56	*0.955*
	SD	9.70	9.88		9.65	9.36	11.09	7.81	

**Table 4:** Bilateral asymmetry. The presence of differences between patients limb sides are presented here with individuals divided into three groups based on the magnitude of difference between the two sides, irrespective of side.

Parameter / differences	Under 3 mm/degree (n=)	Between 3 and 5 mm/degree (n=)	More than 5 mm/degree (n=)
Femoral head diameter	319	1	0
Femoral offset	266	23	31
Neck length	274	20	26
*Neck shaft angle*	*252*	*39*	*29*
*FM-FS*	*318*	*2*	*0*
Femoral torsion	206	22	92

## Data Availability

The data used to support the findings of this study are available from the corresponding author upon request.
